# Interobserver Variability in Lung-RADS Categorization Between Tertiary and Non-Tertiary Hospitals, and Its Implications for Patient Management

**DOI:** 10.3390/jcm15176863

**Published:** 2026-09-04

**Authors:** You Na Kim, Seulgi You, Joo Sung Sun, Kyung Joo Park, Seohyeon Park, Taesun Han, Young Keun Sur

**Affiliations:** 1 Department of Radiology, Ajou University School of Medicine, Suwon 16499, Republic of Korea; dbsk93@gmail.com (Y.N.K.); sunnahn@ajou.ac.kr (J.S.S.); kjpark@ajou.ac.kr (K.J.P.); 2 Department of Statistics, Korea University, Seoul 02841, Republic of Korea; seohyeon20@korea.ac.kr; 3 Department of Radiology, Texas Tech University Health Sciences Center El Paso, El Paso, TX 79905, USA; tshan07@gmail.com; 4 Health Promotion Center, Dankook University Hospital, Cheonan 31116, Republic of Korea; youngkeunsur@gmail.com

**Keywords:** Lung-RADS, lung cancer, low-dose computed tomography, lung cancer screening, observer variability

## Abstract

**Background/Objectives**: Lung-RADS was introduced to standardize the management of nodules detected during lung cancer screening. However, significant interobserver variability has been reported, and its consistency across different hospital settings remains insufficiently investigated. This study aimed to compare Lung-RADS categorization and diagnostic performance between tertiary and non-tertiary hospitals, and to evaluate interobserver variability and associated differences in recommended management. **Methods**: This single-tertiary-center, referral-based retrospective study included patients referred to a tertiary hospital after lung cancer screening at 29 non-tertiary hospitals between September 2019 and December 2023. CT images were reinterpreted at a tertiary hospital using Lung-RADS. A blinded review was performed by an independent thoracic radiologist, and interobserver variability was assessed using Cohen’s kappa. Discordance rate and major discordance (≥9-month follow-up difference) were determined. Sensitivity, specificity, and diagnostic accuracy were calculated and compared. **Results**: Sixty patients (median age, 63 years [interquartile range, 58–68]; 59 men and one woman) with 43 confirmed final diagnoses (18 of lung cancer and 25 of benign lesions) were included. Interobserver variability was fair between tertiary and non-tertiary hospitals (κ = 0.30), and between non-tertiary hospitals and thoracic radiologist (κ = 0.28), but almost perfect between tertiary hospital and thoracic radiologist (κ = 0.82). Lung-RADS discordance occurred in 50.0% of the cases (30/60), with 70.0% (21/30) classified as major discordance. Among the 43 patients with confirmed final diagnoses, specificity was significantly higher in tertiary hospital reports (68.0%, 95% CI 46.5–85.1) compared to non-tertiary hospital reports (20.0%, 95% CI 6.8–40.7; *p* = 0.001), while sensitivity was comparable (100.0%, 95% CI 81.5–100 vs. 88.9%, 95% CI 65.3–98.6; *p* = 0.480). **Conclusions**: Frequent discordances in Lung-RADS categorization were observed between tertiary and non-tertiary hospitals. Higher specificity was observed for tertiary hospital interpretations within this referral cohort. These discrepancies may affect patient management, highlighting the need for strategies to improve consistency.

## 1. Introduction

Lung cancer screening (LCS) using low-dose chest CT is effective in reducing lung cancer-specific mortality among high-risk individuals, leading to its implementation in many countries [1,2]. In Republic of Korea, a national LCS program was introduced in 2019 for high-risk individuals aged 54–74 years with a smoking history of ≥30 pack-years [3]. To ensure consistency in nodule management, the American College of Radiology introduced Lung-RADS, which standardizes reporting and follow-up recommendations for lung nodules detected in LCS [4]. A modified version, known as the Korean Lung-RADS, was developed for Republic of Korea [3,5]. The Korean Lung-RADS includes a new category (2b) designed to reduce false positives associated with tuberculous granuloma or intrapulmonary lymph nodes, allowing radiologists to assign it when either condition is suspected, even if the nodule size corresponds to category 3 or 4 [5]. Additionally, category 4X incorporates findings that suggest a high suspicion of lung cancer, such as obstructive pneumonia, consolidation, or lymph node enlargement without a visible lung nodule.

Lung-RADS is effective in standardizing nodule management; however, previous studies reported substantial variability in its application, with kappa values ranging from 0.34 to 0.99. This variability is influenced by various factors, including radiologist experience, method of nodule measurement (manual vs. semi-automatic), measurement metric used (diameter vs. volume), nodule type (solid vs. subsolid), and use of subjective categories such as 4X [6,7,8,9,10,11,12,13,14,15,16]. In Republic of Korea, patients who undergo initial LCS at non-tertiary hospitals are often referred to a tertiary hospital for second-opinion interpretations or procedures such as bronchoscopy or lung biopsy. However, differences between hospital settings may reflect not only institutional characteristics but also radiologist-level factors, including subspecialty training and experience, access to clinical information and interpretation or measurement practices. To our knowledge, no studies have directly compared Lung-RADS categorization between referring and tertiary interpretations or evaluated how such discrepancies may affect subsequent patient management.

Accordingly, this study aims to evaluate interobserver variability in Lung-RADS categorization between non-tertiary and tertiary hospital interpretations, compare their diagnostic performance and access its influence of categorization discrepancies on patient management.

## 2. Materials and Methods

### 2.1. Study Sample

**Figure 1:**
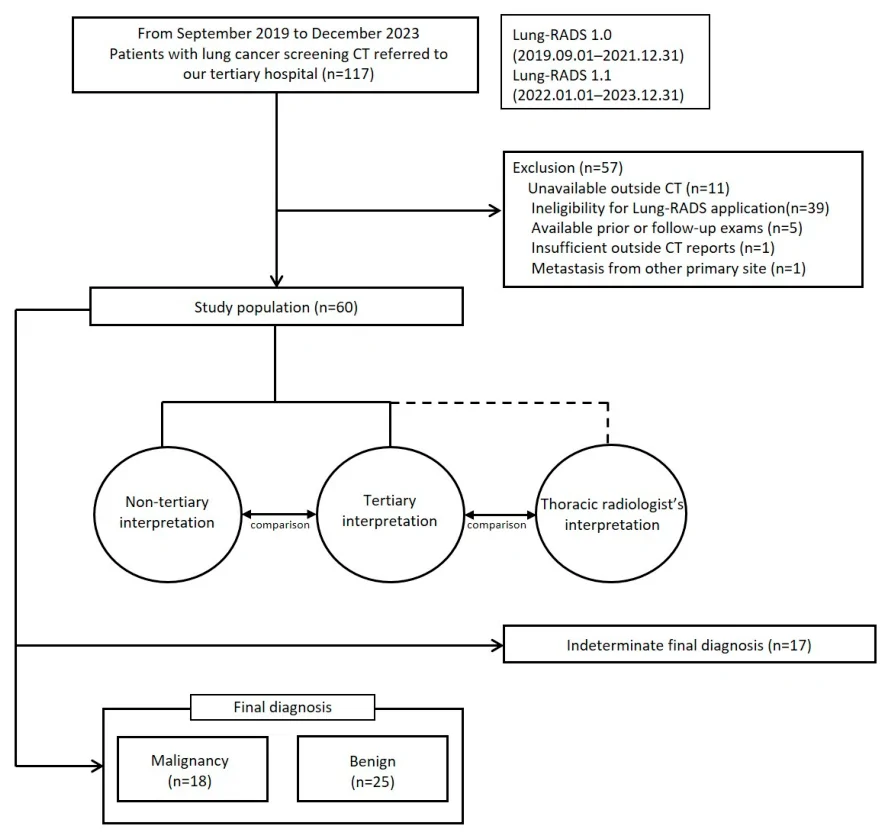
Summary of the flow chart.

This retrospective study was approved by the Institutional Review Board (IRB No. AJOUIRB-DB-2024-394), and the requirement for informed consent was waived. The study was conducted at a single tertiary hospital in Republic of Korea. Using the electronic medical record (EMR) and picture archiving and communication system, cases were identified in which chest CT scans had been performed at non-tertiary hospitals and interpreted by radiologists at those facilities using Lung-RADS between September 2019 and December 2023. In the initial search, 117 patients were identified. Reader 1 (2 years of post-training experience in thoracic radiology) reviewed EMRs from these patients and assessed baseline clinical characteristics, including age, sex, smoking history, and initial CT reports with Lung-RADS categorization from the non-tertiary hospitals. Patients were excluded based on the following criteria: (1) unavailability of external CT images; (2) failure to meet Lung-RADS eligibility criteria (age < 54 or >74 years, or smoking history < 30 pack-years); (3) availability of prior or follow-up examinations (chest CT, chest radiography, or bronchoscopy) at a tertiary hospital before the final CT report, as these examinations could influence Lung-RADS categorization; (4) insufficient external CT reports; and (5) metastasis from other primary sites. Finally, 60 patients from 29 institutions were included (Figure 1).

All CT scans included in this study, obtained from 29 different institutions, were performed using a tube voltage of 100–120 kVp and a volume CT dose index (CTDIvol) of <3.0 mGy. Each scan included thin-section (≤1.25 mm, sharp kernel) and thick-section images (2.5–3 mm), along with one or more multiplanar reformations.

### 2.2. Secondary Interpretations at the Tertiary Hospital and Assessment of Lung-RADS Categories

All CT images were reinterpreted as part of routine clinical practice by one of three randomly assigned thoracic radiologists (readers 2, 3, and 4 with 7, 25, and 37 years of post-training experience in thoracic radiology, respectively). As in routine practice, they had access to clinical and referral information available at the time of interpretation, including the reason for referral, smoking history, and relevant clinical history. Nodule size was manually measured during reinterpretation; however, Lung-RADS categorization was not applied. Based on the reinterpretation reports, the thoracic radiologist (reader 1) retrospectively assigned Lung-RADS categories. To minimize bias, reader 1 was blinded to the original non-tertiary interpretation and subsequent outcomes. Categories were assigned according to the following criteria. First, the risk-dominant nodule or lesion was used to determine the final Lung-RADS category. Second, the nodule type and size were determined based on the reinterpretation report (*n* = 39). When the nodule size was not explicitly documented in the report, reader 1 measured the average diameter in a lung window setting (*n* = 11). For part-solid nodules, the average diameters of both the total nodule and its solid component were measured. Third, if the report suggested an infection rather than a nodule, category S was assigned (*n* = 4). Fourth, if the report indicated a tuberculous granuloma, the nodule was classified as category 2b. Fifth, if no nodules or significant findings were reported, reader 1 re-evaluated the CT images, and category 1 was assigned when no abnormalities were confirmed (*n* = 6). In Republic of Korea, Lung-RADS version 1.0 was used until 2021, after which version 1.1 was adopted for the national LCS program from 2022 to 2023. Therefore, the appropriate Lung-RADS version was applied based on the period in which chest CT was conducted.

### 2.3. Interobserver Agreement in Lung-RADS Categorization

To mitigate potential bias arising from clinical information beyond CT imaging, one of the thoracic radiologists (reader 2) independently reviewed all CT scans and assigned Lung-RADS categories while being blinded to all clinical data, including prior interpretations. To minimize recall bias, a washout period of >6 months was maintained between the initial reinterpretation and Lung-RADS assessment. The reader assessed risk-dominant nodules or lesions and assigned categories using the Lung-RADS version 1.0 for images acquired before 2022 and Lung-RADS version 1.1 for images acquired from 2022 to 2023. Interobserver agreement was evaluated among reports from non-tertiary hospitals, the tertiary hospital, and blinded interpretations by the independent thoracic radiologist.

### 2.4. Assessment of Lung-RADS Categorization Discordance Between Non-Tertiary and Tertiary Hospitals

To assess the influence of categorization variability on patient management, Lung-RADS categories 1 and 2 were grouped as concordant owing to their identical management recommendations; the same grouping was applied to categories 4B and 4X. Discordant cases were categorized into two groups based on the differences in recommended follow-up intervals. Major discordance was defined as a discrepancy involving a follow-up interval difference of ≥9 months (e.g., category 1/2 vs. 4A or 4B/4X), while minor discordance referred to differences of up to 6 months (e.g., category 1/2 vs. 3, 3 vs. 4A, or category 4A vs. 4B/4X) [17]. Furthermore, the underlying causes of discrepancies were examined by determining whether they were attributable to the selection of different risk-dominant nodules, the assignation of different categories to the same risk-dominant nodule, or the interpretation of the same lesion differently (e.g., nodule vs. infection, or endobronchial nodule vs. secretion).

### 2.5. Comparison of Diagnostic Performance: Non-Tertiary vs. Tertiary Interpretations

The diagnostic performance of Lung-RADS interpretations by non-tertiary and tertiary hospitals in predicting lung cancer was evaluated by calculating sensitivity, specificity, accuracy, positive predictive value (PPV), and negative predictive value (NPV). The reference standard for this evaluation was established as follows [18]: lesions were classified as malignant if confirmed by pathological examination through biopsy or surgical resection. The lesions were considered benign based on the following criteria: (1) stability of solid nodules for ≥2 years, (2) stability of subsolid nodules for ≥5 years, (3) interval regression on serial CT scans, or (4) histopathologic confirmation of benignity [19]. Patients in whom no nodules were detected in tertiary interpretation and follow-up CT images were considered benign for diagnostic performance analysis. Solid nodules with less than 2 years of follow-up and subsolid nodules with less than 5 years were classified as indeterminate and excluded from the diagnostic performance analysis [19].

### 2.6. Statistical Analysis and AI Usage

Interobserver agreement was assessed among the results of non-tertiary interpretation, tertiary interpretation, and the reinterpretation by the thoracic radiologists. Fleiss’ kappa statistic was used to measure agreement across all interpretations. For pairwise comparisons, Cohen’s kappa was used. Kappa values were interpreted according to the following scale: poor (<0.00), slight (0.00–0.20), fair (0.21–0.40), moderate (0.41–0.60), good (0.61–0.80), and almost perfect (0.81–1.00) [20]. For diagnostic performance comparison across interpretations, accuracy, sensitivity and specificity were assessed using the McNemar test with continuity correction, while PPV and NPV were compared using Park’s permutation test [21]. Each performance metric was evaluated based on positive screening results, defined as category 3 or higher [22]. All statistical analyses were performed using MedCalc (version 22; MedCalc Software Ltd., Ostend, Belgium) and R software (version 4.2.2; R Foundation for Statistical Computing, Vienna, Austria). Statistical significance was set at *p* < 0.05. During the preparation of this work the authors used Claude Sonnet 5 (Anthropic, San Francisco, CA, USA) in order to refine the English phrasing and grammar of the manuscript.

## 3. Results

### 3.1. Patient Characteristics

Table 1 shows a summary of the patient demographics and final diagnoses. Overall, 59 men (98.3%) and one woman (1.7%), with a median age of 63 years (interquartile range [IQR], 58–68) and a median smoking history of 40 pack-years (IQR, 34–49), were included. Among the 29 referring non-tertiary hospitals, 21 institutions contributed 1 case each, 4 contributed 2 cases, and 1 institution each contributed 3, 5, 11, and 12 cases. The reasons for referral from non-tertiary hospitals were as follows: 49 solid nodules, three part-solid nodules, two ground glass nodules, four airway nodules, and two suspected infections. The final diagnosis was confirmed in 43 patients, excluding nine patients who remained under follow-up and eight patients who were lost to follow-up. Among the 43 patients with a final diagnosis, 18 were diagnosed with lung cancer and 25 with benign lesions. Compared with patients with a confirmed diagnosis, those with indeterminate outcomes did not differ significantly in age, smoking history, Lung-RADS version, or the proportion of positive non-tertiary interpretations. However, positive tertiary interpretations were significantly less frequent among indeterminate cases (17.6% vs. 60.5%, *p* = 0.004);

### 3.2. Interobserver Agreement for Lung-RADS Categorization

The overall interobserver agreement for the Lung-RADS categorization was moderate, with a Fleiss’ kappa value of 0.44 (95% CI: 0.36–0.56). Pairwise comparisons indicated fair agreement between non-tertiary and tertiary interpretations (κ = 0.30, 95% CI 0.16–0.48), as well as between non-tertiary interpretations and those of the thoracic radiologist (κ = 0.28, 95% CI 0.13–0.43). In contrast, an almost perfect agreement was observed between interpretations from the tertiary hospital and those of the thoracic radiologist (κ = 0.82, 95% CI 0.70–0.94). In the sensitivity analysis stratified by Lung-RADS version, the pattern of agreement was similar for version 1.0 (*n* = 25) and version 1.1 (*n* = 35). Agreement between non-tertiary and tertiary interpretations was fair for both versions (κ = 0.32, 95% CI 0.12–0.53 and κ = 0.28, 95% CI 0.11–0.46, respectively), as was agreement between non-tertiary interpretations and those of the thoracic radiologist (κ = 0.29, 95% CI 0.09–0.51 and κ = 0.22, 95% CI 0.06–0.39, respectively). In contrast, agreement between tertiary interpretations and those of the thoracic radiologist remained good to almost perfect (κ = 0.84, 95% CI 0.66–1.00 and κ = 0.77, 95% CI 0.59–0.92) (Supplementary Table S1).

### 3.3. Discordance in Lung-RADS Categorization Between Non-Tertiary and Tertiary Hospitals

The discordance rate between non-tertiary and tertiary interpretations was 50.0% (30 of 60 cases). Table 2 shows the distribution of the final Lung-RADS categories assigned in non-tertiary and tertiary interpretations. Among the 30 discordant cases, the major and minor discordance rates based on recommended follow-up intervals were 70.0% (21/30) and 30.0% (9/30), respectively. As categories 3 and 4 were considered positive screening results, non-tertiary hospital interpretations showed a higher proportion of positive screening results (50/60, 83.3%) than that of the tertiary hospital (29/60, 48.3%).

**Figure 2:**
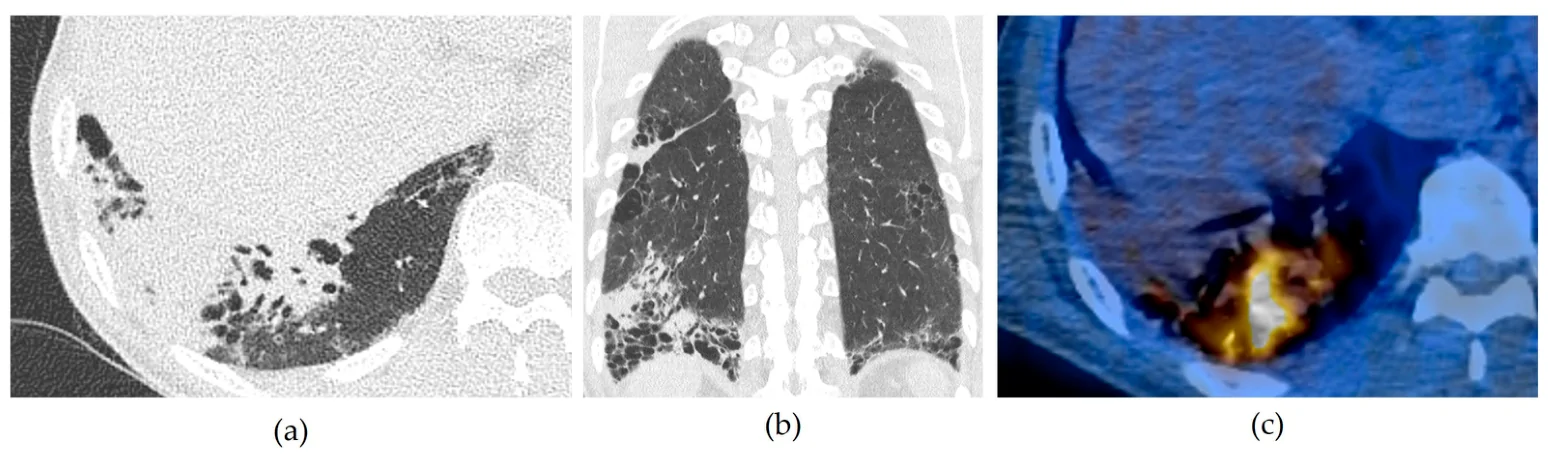
Chest CT images of a 70-year-old man with a 50-pack-year smoking history. (**a**,**b**) CT images show a consolidative mass in the right lower lobe, with coexisting usual interstitial pneumonia pattern interstitial lung disease characterized by honeycombing and traction bronchiectasis in both lower lobes. A non-tertiary hospital radiologist categorized the lesion as S (suggestive of pneumonia), whereas a thoracic radiologist at a tertiary hospital assigned a Lung-RADS category 4X. (**c**) The fluorodeoxyglucose (FDG) PET/CT image shows intense FDG uptake in the RLL mass. The lesion was ultimately confirmed as squamous cell carcinoma on lung biopsy.

Of the 60 referred cases, 26 (43.3%) were downgraded, and 4 (6.7%) were upgraded in the tertiary interpretations. Among the 21 cases with major discordance, 19 were downgraded from category 4A (*n* = 12) or 4B/4X (*n* = 7) to category 1/2, none of which had been diagnosed with lung cancer during the available follow-up. These discrepancies could have resulted in more intensive follow-up or diagnostic evaluation based on the initial non-tertiary interpretations. In contrast, two major discordant cases were initially interpreted as infections in non-tertiary interpretations but were later confirmed as lung cancer (Figure 2). The other two were upgraded from category 4A to 4X; one was confirmed as lung cancer, and the other as a benign lesion. Furthermore, seven cases (11.7%) were downgraded from category 4B or 4X to negative, substantially altering their management plans. Among these seven cases, four nodules were confirmed as benign, and the rest remained under follow-up.

**Figure 3:**
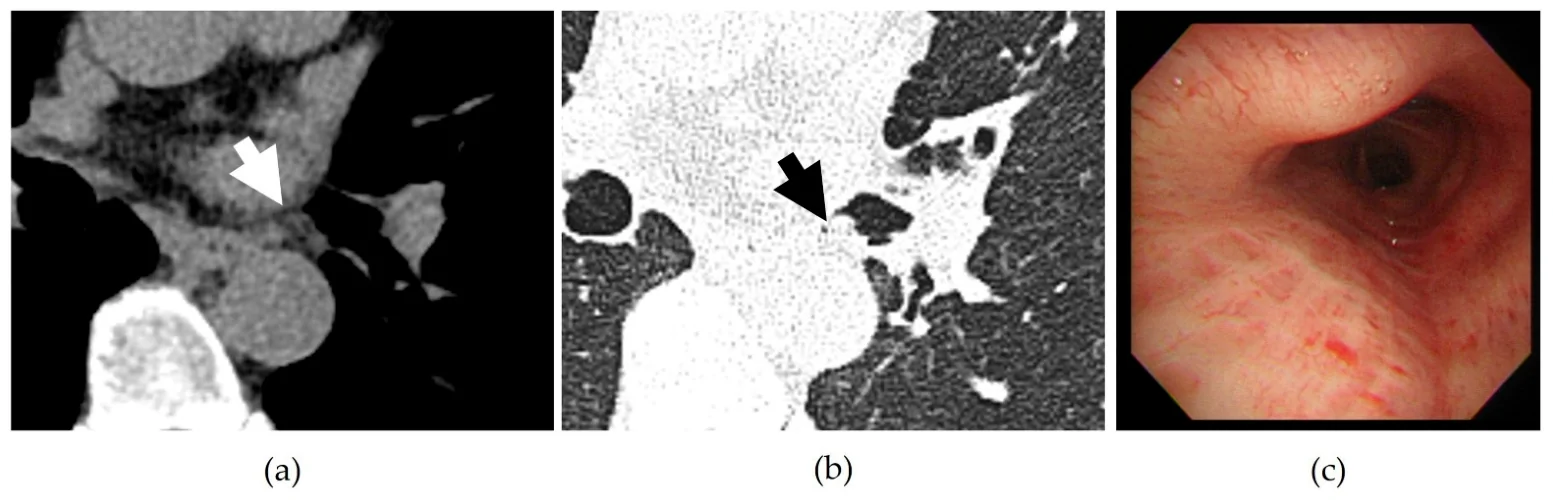
Chest CT images of a 63-year-old man with a 40-pack-year smoking history. (**a**) The mediastinal window CT image shows an oval-shaped, low-density lesion (arrow) in the left main bronchus. (**b**) Axial CT image in lung window setting with 1 mm slice thickness reveals an internal micro air bubble (arrow) within the lesion. A non-tertiary hospital radiologist categorized the lesion as Lung-RADS category 4A (suggestive of an endobronchial nodule). However, a thoracic radiologist at a tertiary hospital categorized it as category 1 (secretion). (**c**) Bronchoscopy revealed no evidence of an endobronchial lesion.

None of the 30 discordant cases resulted from selecting different risk-dominant nodules. In 20 cases (66.7%), discrepancies arose from assigning different categories to the same risk-dominant nodule, while in the remaining 10 cases (33.3%), they were attributed to different interpretations of the same lesion. Further details are presented in Table 3. Among the 20 cases with discordant categorization of the same nodule, size measurements differed in 11 cases. In the remaining nine cases, nodules were categorized differently based on their characteristics other than size: six were identified as tuberculosis granulomas (category 2b), one as a hamartoma (category 1), and two as category 4X in tertiary interpretations. The different interpretation for the same lesions resulted from the following reasons: (1) two lesions initially interpreted as infections in non-tertiary hospitals were later classified as category 4X based on tertiary hospital interpretations and confirmed pathologically as lung cancer; (2) four cases initially interpreted as nodules in non-tertiary hospitals—including one part-solid nodule and one ground glass nodule—were subsequently interpreted as infections at the tertiary hospital; and (3) four cases categorized as endobronchial nodules in non-tertiary hospitals were reinterpreted as secretions in the tertiary hospital (Figure 3).

### 3.4. Comparison of the Diagnostic Performance Between Non-Tertiary and Tertiary Lung-RADS Categorization for Malignancy Prediction

Table 4 shows the diagnostic performance of Lung-RADS categorization for lung cancer diagnosis. The non-tertiary interpretations yielded a sensitivity, specificity, and accuracy of 88.9% (16/18, 95% CI 65.3–98.6), 20.0% (5/25, 95% CI 6.8–40.7), and 48.8% (21/43, 95% CI 33.3–64.5), respectively. In contrast, tertiary interpretations demonstrated a sensitivity, specificity, and accuracy of 100% (18/18, 95% CI 81.5–100), 68.0% (17/25, 95% CI 46.5–85.1) and 81.4% (35/43, 95% CI 66.6–91.6), respectively. Whereas sensitivity did not significantly differ between the two settings, specificity was significantly higher for tertiary hospital interpretations than for those from non-tertiary hospitals (68.0% vs. 20.0%, *p* = 0.001).

To assess the potential influence of patients with indeterminate outcomes, diagnostic performance was recalculated under two extreme assumptions (Supplementary Table S2). When all 17 indeterminate cases were assumed benign, specificity was 19.0% (95% CI 8.6–34.1) for non-tertiary interpretations and 73.8% (95% CI, 58.0–86.1) for tertiary interpretations, and sensitivity was unchanged. When all were assumed malignant, the corresponding specificities were 20.0% (95% CI 6.8–40.7) and 68.0% (95% CI 46.5–85.1), respectively. However, the sensitivity of tertiary interpretations decreased to 60.0% (95% CI 42.1–76.1) and was lower than that of non-tertiary interpretations (85.7%, 95% CI 69.7–95.2; *p* = 0.027).

## 4. Discussion

As a standardized system, Lung-RADS requires consistent interobserver reliability to ensure appropriate patient management. In real-world clinical practice, second-opinion imaging interpretations are commonly sought to ensure appropriate clinical decisions or upon request by patients [23,24]. This study evaluated variability in Lung-RADS categorization of LCS CT scans between non-tertiary and tertiary hospitals. Interobserver agreement between the two settings was fair (κ = 0.30). Lung-RADS discordance occurred in 50.0% of cases, and 70.0% of the discordant cases (21/30) were major discordance. Non-tertiary hospitals reported more positive screening results (83.3%) than tertiary hospitals (48.3%). Furthermore, specificity for lung cancer detection was significantly higher in tertiary hospitals (68.0%) than in non-tertiary hospitals (20.0%).

Several studies have reported moderate-to-substantial interobserver agreement for Lung-RADS categorization [25]. However, our findings reveal only a fair agreement between non-tertiary and tertiary interpretations, whereas agreement between the interpretation by the tertiary hospital and the independent thoracic radiologist was almost perfect. The tertiary interpretations were performed by experienced thoracic radiologists, whereas information regarding the subspecialty and experience of radiologists at the referring hospitals was unavailable. Therefore, the observed differences cannot be attributed solely to the hospital setting and may also reflect differences in radiologist expertise or interpretation practices.

The high positive-screen rate (83.3%) in non-tertiary hospital interpretations should be interpreted in the context of this referral-based cohort, as patients with positive or suspicious findings were more likely to be referred to the tertiary hospital. This referral pattern may have contributed to the observed positive-screen rate and diagnostic performance, limiting the generalizability of these findings to unselected lung cancer screening populations. Similarly, although tertiary interpretations showed 100% sensitivity, this estimate was based on only 18 malignant cases with a wide confidence interval (95% CI 81.5–100), and it should not be interpreted as evidence of definitive superiority. Differences in interpretation practices may also have contributed to the observed discordance. Radiologists at initial screening centers may prioritize sensitivity to avoid missing potential malignancies, whereas thoracic radiologists at tertiary hospitals may apply stricter criteria. However, such differences in interpretation strategies were not directly assessed in this study and should be considered a possible explanation. Regardless of the underlying cause, differences in Lung-RADS categorization may result in differences in recommended follow-up or diagnostic evaluation. Comprehensive training programs and continuing education for participating radiologists could be considered potential strategies to improve consistency in Lung-RADS categorization and reduce interobserver variability. Regarding the influence of interobserver disagreement on patient management, Van Riel et al. have reported that major discordance, leading to a ≥9-month difference in follow-up time, occurred in 8% of cases [17]. In contrast, our findings revealed a higher rate of major discordance (35.0%, 21/60) in the overall cohort. Three of the four cases upgraded to category 4X were pathologically confirmed as lung cancer, suggesting that such reclassification may prompt earlier diagnostic evaluation in some patients. Seven cases (7/60, 11.7%) were downgraded from category 4B or 4X to negative, including four that were confirmed as benign. Such downgrading could reduce unnecessary short-interval imaging or tissue sampling. These findings suggest that discrepancies in Lung-RADS categorization may have substantial implications for patient management in the clinical referral setting.

Inconsistent size measurements for the same nodule accounted for the largest proportion of discordant cases (36.7%, 11/30). At our tertiary hospital, average diameters were manually measured, whereas the specific methods used by non-tertiary hospitals (whether manual or semi-automated) were unknown. However, semi-automated measurements may improve interobserver agreement compared to measurement by manual methods [25]. Therefore, a wider implementation of semi-automated measurement tools may help reduce categorization discrepancies caused by measurement variability. Subjective categories, such as Korean Lung-RADS 2b (20.0%, 6/30) and 4X (13.3%, 4/30), accounted for a substantial proportion of disagreements. Because these categories are influenced by reader expertise, dedicated Lung-RADS training with feedback, structured reporting, and decision-support tools incorporating Lung-RADS criteria may help improve consistency and reduce interobserver variability [12].

This study has some limitations. First, it was a retrospective, single-center study with a small sample size, including only 18 patients with malignancy, which limited the statistical power of the diagnostic performance analysis. Moreover, diagnostic accuracy was evaluated only in those cases with a confirmed final diagnosis (43 out of 60). Because the indeterminate cases were predominantly those categorized as negative at the tertiary hospital, this differential verification may have favored the specificity of tertiary interpretations, and the exclusion of these patients limited the reliability and generalizability of the diagnostic performance estimates. Second, referral bias may have occurred, as most of the cases included had been initially categorized as positive at non-tertiary hospitals, whereas many negative cases may not have been referred to the tertiary hospital. Additionally, potential clustering by referring institution was not accounted for in the statistical analyses. Third, Lung-RADS categories for tertiary hospital interpretations were retrospectively reconstructed based on the original radiology reports rather than assigned contemporaneously at the time of interpretation. Although reader 1 was blinded to the original non-tertiary Lung-RADS categories and subsequent outcomes during this process, these different assessment conditions may have introduced reconstruction bias. In addition, two versions of Lung-RADS (versions 1.0 and 1.1) were also applied according to the study period, which may have contributed to heterogeneity in categorization. Fourth, many discordances resulted from differences in nodule size measurements; however, we could not confirm whether manual or semi-automated methods were used for this purpose. Therefore, size-related discordance may reflect differences in measurement protocol rather than interpretation. Fifth, information regarding the subspecialty and experience of radiologists at the non-tertiary hospitals was unavailable. Therefore, we could not determine whether the observed differences were attributable to the hospital setting itself or to differences in radiologist expertise. Finally, CT scanning protocols and equipment varied across institutions. All scans were performed following the guidelines of the Korean National Lung Cancer Screening Program, and a low-dose protocol was followed, making significant influence on outcomes unlikely.

## 5. Conclusions

In conclusion, substantial discordance in Lung-RADS categorization was observed between tertiary and non-tertiary hospitals. Within this referral cohort, lower specificity for lung cancer detection was observed for non-tertiary hospital interpretations. Given that interobserver disagreement may affect patient management, continuous education and feedback may help consistency and optimize decision-making in LCS.

## Figures and Tables

**Table 1:** Summary of patient characteristics and follow-up results.

Variable	Total (*n* = 60)
Age (years) *	63 (58–68)
Sex	
Male	59
Female	1
Smoking	
Pack-year *	40 (34–49)
Uncertain value	6
Final diagnosis	
Malignancy	
Lung cancer	18
Benign	
Benign nodules	20
Secretions	4
No detectable nodule	1
Indeterminate	17

* Data are medians, with interquartile range in parentheses.

**Table 2:** Comparison of Lung-RADS categories between non-tertiary and tertiary interpretations.

	Tertiary Interpretation				
Non-tertiary interpretation		1/2(2b)	3	4A	4B/4X
	1/2(2b)	8	0	0 *	2 *
	3	4	1	0	0
	4A	12 *	2	6	2
	4B/4X	7 *	0	1	15

Matrix illustrating agreement and discordance in Lung-RADS categories between non-tertiary hospitals and a tertiary hospital. Diagonal cells represent concordant categorizations. Off-diagonal cells indicate discordant cases. Asterisks (*) denote major discordance, defined as a ≥9-month difference in the recommended follow-up interval.

**Table 3:** Causes of disagreement in Lung-RADS category assessment.

Reasons for Observer Disagreement	Number (%)
Different risk-dominant nodule	0 (0.0)
Same risk-dominant nodule	20 (66.7)
Different nodule size measurement	11 (36.7)
Different category for the same nodule	9 (30.0)
Tuberculosis granulomas	6 (20.0)
Hamartoma	1 (3.3)
Upgrade to 4X	2 (6.7)
Different interpretation for the same lesion (non-tertiary vs. tertiary)	10 (33.3)
Infection vs. lung cancer	2 (6.7)
Nodule vs. infection	4 (13.3)
Endobronchial nodule vs. secretion	4 (13.3)
Total	30

**Table 4:** Diagnostic performance of non-tertiary and tertiary hospitals for Lung-RADS in predicting lung cancer.

Positive Screening	Sensitivity (%) [95% CI]	Specificity (%) [95% CI]	Accuracy (%) [95% CI]	PPV (%) [95% CI]	NPV (%) [95% CI]
Non-tertiary interpretations	88.9 (16/18) [65.3, 98.6]	20.0 (5/25) [6.8, 40.7]	48.8 (21/43) [33.3, 64.5]	44.4 (16/36) [27.9, 61.9]	71.4 (5/7) [29, 96.3]
Tertiary interpretations	100.0 (18/18) [81.5, 100]	68.0 (17/25) [46.5, 85.1]	81.4 (35/43) [66.6, 91.6]	69.2 (18/26) [48.2, 85.7]	100.0 (17/17) [80.5, 100]
*p*-Value	0.48 *	0.001 *	<0.001 *	<0.001 ^†^	<0.001 ^†^

* Comparison of sensitivity, specificity and accuracy: McNemar’s test with continuity correction. ^†^ Comparison of PPV and NPV: Park’s permutation test. CI = confidence interval; PPV = positive predictive value; NPV = negative predictive value.

## Data Availability

The data presented in this study are available upon request from the corresponding author. The data are not publicly available due to privacy and ethical restrictions.

## Supplementary Materials

The following supporting information can be downloaded at https://www.mdpi.com/article/10.3390/jcm15176863/s1: Table S1. Interobserver agreement for Lung-RADS categorization, overall and stratified by Lung-RADS version; Table S2. Diagnostic performance under alternative assumptions for the 17 patients with indeterminate outcomes.

## Abbreviations

The following abbreviations are used in this manuscript: LCS, lung cancer screening; EMR, electronic medical record; PPV, positive predictive value; NPV, negative predictive value; IQR, interquartile range.
