# Supplementary material for: Interobserver Variability in Lung-RADS Categorization Between Tertiary and Non-Tertiary Hospitals, and Its Implications for Patient Management
Source: J Clin Med. 2026 Sep 4;15(17):6863. doi: 10.3390/jcm15176863 (PMC13565784; doi:10.3390/jcm15176863)
Supplement: Supplementary file 1 [file jcm-15-06863-s001.zip › jcm-4530094-supplementary.pdf]

Supplementary Table S1. Interobserver agreement for Lung-RADS categorization, overall and stratified by Lung-RADS version

Comparison	Overall (n = 60)	version 1.0 (n = 25)	version 1.1 (n = 35)
All readers, Fleiss' κ	0.44 (0.36, 0.56)		
Non-tertiary vs. Tertiary	0.30 (0.16, 0.48)	0.32 (0.12, 0.53)	0.28 (0.11, 0.46)
Non-tertiary vs. Thoracic radiologist	0.28 (0.13, 0.43)	0.29 (0.09, 0.51)	0.22 (0.06, 0.39)
Tertiary vs. Thoracic radiologist	0.82 (0.70, 0.94)	0.84 (0.66, 1.00)	0.77 (0.59, 0.92)

Data are unweighted Cohen's κ (95% CI) unless otherwise indicated. Version 1.0 was applied to CT scans obtained before 2022 and version 1.1 to scans obtained from 2022 to 2023.

Supplementary Table S2. Diagnostic performance under alternative assumptions for the 17 patients with indeterminate outcomes.

Assumption	Interpretations	Sensitivity (%) [95% CI]	Specificity (%) [95% CI]	Accuracy (%) [95% CI]	PPV (%) [95% CI]	NPV (%) [95% CI]
All benign	Non-tertiary	88.9 (16/18) [65.3, 98.6]	19.0 (8/23) [8.6, 34.1]	40.0 (24/60) [27.6, 53.5]	32.0 (16/50) [19.5, 46.7]	80.0 (8/10) [44.4, 97.5]
	Tertiary	100 (18/18) [81.5, 100]	73.8 (31/42) [58.0, 86.1]	81.7 (49/60) [69.6, 90.5]	62.1 (18/26) [42.3, 79.3]	100 (31/31) [88.8, 100]
	p-value	0.48 *	<0.001 *	<0.001 *	<0.001 †	<0.001 †
All malignant	Non-tertiary	85.7 (30/35) [69.7, 95.2]	20.0 (5/25) [6.8, 40.7]	58.3 (35/60) [44.9, 70.9]	60.0 (30/50) [45.2, 73.6]	50.0 (5/10) [18.7, 81.3]
	Tertiary	60.0 (21/35) [42.1, 76.1]	68.0 (17/25) [46.5, 85.1]	63.3 (38/60) [49.9, 75.4]	72.4 (21/29) [52.8, 87.3]	54.8 (17/31) [36.0, 72.7]
	p-value	0.027 *	0.001 *	0.689 *	0.064 †	0.7 †

*Comparison of Sensitivity, Specificity and Accuracy: McNemar's test with continuity correction.

†Comparison of PPV and NPV: Park's permutation test. CI = confidence interval; PPV = positive predictive value; NPV = negative predictive value.
